# Practical Medicine

**Published:** 1858-05

**Authors:** 


### Bi-Monthly Periscope.

### PRACTICAL MEDICINE.

I. On Emboli. By *Professor Schutzenberger.*—The following are the principal conclusions of an elaborate series of papers, published by Professor Schutzenberger, of Strasburg, upon the obstruction of arteries by fibrinous clots detached from the heart or large vessels.

1. Fibrinous concretions or solid bodies formed in the heart or large arteries may become detached from their primary seat, be carried into the torrent of the circulation, and obstruct different secondary branches of the arterial tree. The circumstance is neither absolutely rare nor exceptional, and it constitutes a special and very remarkable affection of arteries to which Virchow has given the name of arterial emboli. 2. The existence of the affection is proved, (*a*) By scientific induction, which demonstrates the possibility of the formation of solid bodies in the heart and large vessels, and the probability that such bodies may become free and floating; and by experiment, which proves that the sanguineous current may carry them to a distance. (*b*) By numerous and conclusive clinical and microscopical observations, agreeing among themselves and admitting of no other interpretation. (*c*) By microscopic researches, which have shown in some cases the speciality and identity of certain of these bodies simultaneously observed in the heart and in the plugs obstructing the arteries. 3. Arterial emboli is then a real and very serious disease, not of infrequent occurrence, but long misunderstood. It should be admitted into the nosological scheme, and deserves all the attention of the clinical observer and the pathological anatomist. 4. It has been observed as a consequence of (1) Gangrenous phlebitis of the pulmonary veins. (2) Of organic affections of the left side of the heart; and of (3) The atheromatous degeneration of the large arterial trunks. Its most frequently observed cause is found in the fibrinous, or calcareous concretions, or in the polypiform excrescences developed on the mitral valve, and carried away by the circulating torrent. 5. The form, size, consistence, color, and nature of these obturating bodies are variable and notably differ, according to whether the obstruction examined be old or recent. In the former case they may have undergone transformations which may render them unrecognizable; while in the latter, their fibrinous, calcareous, verrucous nature may be shown. 6. When the patient does not succumb to a first arterial obstruction, generally others are produced, the multiplicity and succession of the arterial lesions being one of the characters of the disease. Multiple obturation may be successively produced from the periphery toward the heart, either in various points of the same arterial branch, or in different arterial divisions. 7. The arteries which have been most frequently found obstructed are the subclavian, the internal carotid, the arteries of the lower and upper extremities, the splenic and renal arteries, the external carotid, the mesenterics, etc. 8. The obturation is ordinarily produced at the point of narrowing of an arterial branch immediately below the bifurcation, or the point of departure from a large branch, in the points where the artery incurvates or traverses aponeurotic or bony canals. 9. At first the arterial coats are healthy, and the plug is surrounded by a recent coagulum, which extends upward to the next collateral. Below, the artery may be empty, or may contain recent coagula. The plugs differ from secondary coagula by their color, consistence, and composition. As a consequence of arterial obturation the outer coat may inflame consecutively. The plug contracts adhesions with the inner coats, and the whole is transformed into a ligament-like tissue. 10. If, after the obstruction, a sufficient collateral circulation be set up, the lesion remains localized, and only gives rise to temporary functional disturbance. But if such collateral circulation be not set up, or only imperfectly, consecutive changes are produced in the organs to which the artery is distributed. In the case of obturation of the arteries of the extremities, in the absence of collateral circulation, mortification, or gangrene, general or partial, dry or humid, may be produced. In parenchymatous organs very exactly circumscribed sanguineous or fibrinous infarctus are produced. In the brain, this infarctus usually gives rise to yellow ramollissement, and it is highly probable that certain circumscribed indurations are due to obturation of the arterial ramuscules. In the spleen and the kidneys the infarctus constitutes an entirely special lesion, generally of a conical form, exactly limited, of different color according to its age, and frequently more dense than the remainder of the parenchyma. It is probable that emboli of the small arteries may give rise to other, as yet little known lesions of the parenchymatous organs. 11. The symptoms vary according to the arteries obstructed. Obturation of the cerebral arteries gives rise to disturbance of function analogous to an attack of apoplexy. The symptoms do not differ from those attendant upon cerebral hemorrhage or acute ramollissement. Emboli of the arteries of the limbs is indicated by feelings of numbness, pricking, painful shootings in the limbs, and cessation of the beating of the arteries. These symptoms may disappear if a collateral circulation becomes established, but when this is not the case the ulterior symptoms are those of gangrene. 12. The treatment of the affection is at present only palliative and symptomatic.—*Gaz. des Hôp.*, 1857. No. 80.—*Med. Times & Gazette*, March 18, 1858.

II. VALERIANATE OF AMMONIA SUCCESSFULLY USED IN NEURALGIA.—The efficiency of the valerianate of ammonia as a remedy in the cure of neuralgia, has been frequently proved in a number of patients admitted at the Royal Free Hospital, under the care of Dr. O'Connor. The following case, of twenty-one years' almost constant suffering, is an excellent illustration of the success attending its use:—E. W., a woman of color, thirty-eight years of age, single, by occupation a dress-maker, was admitted an out-patient, under the care of Dr. O'Connor, on the 24th of February last. She stated that since the age of seventeen she had been a constant sufferer from a neuralgic affection of the left side of the head, face, and neck, from which she was seldom free for twelve hours at a time, and her sufferings were often so great as to make life feel a burden to her. She had been under the care of many medical men, and had, without benefit, obtained advice at nearly all the medical institutions of the metropolis. Large doses of quinine and iron, as well as the internal and external use of belladonna and other anodynes, with hydrocyanic acid, were had recourse to without any good result. When the patient presented herself at the Royal Free Hospital she was suffering excruciating pain over the whole of the left cheek, there was dropping of the left eyelid; otherwise she was in good health, and from the age of fourteen had regularly menstruated; there was no evidence of deranged stomach, and she had never suffered from any other illness. These decayed stumps were removed. The dose of the valerianate of ammonia was increased from one drachm to one drachm and a half three times a day. On the 10th of March she was entirely relieved.—*Med. Times and Gazette*, April, 1858.

